# Investigation of the Effect of Preliminary Mechanical Treatment on the Mechanical Properties of 12Kh18N10T Stainless Steel After Ion-Plasma Nitriding

**DOI:** 10.3390/ma19101960

**Published:** 2026-05-10

**Authors:** Zarina Aringozhina, Bauyrzhan Rakhadilov, Arnur Askhatov, Meruyert Adilkanova, Nurtoleu Magazov

**Affiliations:** 1 International School of Engineering, D. Serikbayev East Kazakhstan Technical University, Ust-Kamenogorsk 070010, Kazakhstan; zaringozhina@edu.ektu.kz (Z.A.); madylkanova@ektu.kz (M.A.); magazovn@gmail.com (N.M.); 2 PlasmaScience LLP, Ust-Kamenogorsk 070010, Kazakhstan; rakhadilovb@gmail.com

**Keywords:** 12Kh18N10T stainless steel, ion-plasma nitriding, ultrasonic nanocrystalline surface modification, expanded austenite, microhardness, scratch resistance, surface roughness, nitrogen diffusion

## Abstract

This study investigates the influence of preliminary severe plastic deformation on the efficiency of ion-plasma nitriding (IPN) and the formation of a nitrided layer in 12Kh18N10T austenitic stainless steel. Two types of surface mechanical treatment were compared: vibro-impact ball mechanical treatment (VIMT) and ultrasonic nanocrystalline surface modification (UNSM). After the preliminary treatments, the samples were subjected to ion-plasma nitriding at 500 °C for 10 h using ammonia (NH_3_) as the working gas. The phase composition, microstructure, elemental distribution, surface roughness, microhardness, and scratch resistance were analyzed using X-ray diffraction (XRD), scanning electron microscopy (SEM) with energy-dispersive X-ray spectroscopy (EDS) analysis, profilometry, instrumented indentation, and progressive scratch testing. The results show that both types of preliminary treatment promote the formation of a nitrogen-enriched diffusion layer. The UNSM-treated samples exhibited more pronounced peak broadening and shifting in XRD patterns, indicating a higher level of lattice distortion and nitrogen supersaturation. The maximum nitrogen concentration in the near-surface region reached 15.56 wt.%. Microhardness increased significantly after nitriding for both treatments. Under the selected processing conditions, the UNSM + IPN samples demonstrated a thicker diffusion layer, lower surface roughness, and higher critical loads in scratch testing, indicating improved resistance to surface damage compared with VIMT + IPN samples. The obtained results highlight the important role of the defect structure formed during preliminary treatment in controlling nitrogen diffusion and the resulting mechanical and tribological properties of ion-plasma nitrided austenitic stainless steel.

## 1. Introduction

Austenitic stainless steels are widely used engineering materials due to their excellent corrosion resistance, ductility, and structural stability. Among them, AISI 304 is the most commonly used grade due to its balanced mechanical properties and cost-effectiveness [1]. However, for applications involving elevated temperatures and aggressive environments, more stable alloys such as AISI 321 (12Kh18N10T) are preferred. The addition of titanium stabilizes the austenitic structure by preventing chromium carbide precipitation, thereby improving resistance to intergranular corrosion and enhancing high-temperature performance [1]. Despite these advantages, austenitic stainless steels exhibit relatively low surface hardness and limited wear resistance, which significantly restrict their performance under friction and contact loading conditions [2]. Therefore, the development of effective surface strengthening methods without deterioration of corrosion resistance remains an important task in modern materials science. One of the most promising approaches for surface modification of austenitic steels is ion-plasma nitriding (IPN) [3]. During low-temperature nitriding (typically 350–450 °C), expanded austenite (γN or S-phase) is formed in the surface layer as a supersaturated solid solution of nitrogen in the face-centered cubic (FCC) lattice [4,5,6]. This phase is associated with a significant increase in microhardness, the development of compressive residual stresses, and improved wear resistance while maintaining corrosion resistance [7,8]. The kinetics of γN layer formation is governed by nitrogen diffusion, which strongly depends on the defect structure of the near-surface layer [9,10]. An increased dislocation density, grain refinement, and the presence of subgrain boundaries create accelerated diffusion paths, thereby enhancing nitrogen diffusion and saturation of the material [11]. In addition, residual stresses and structural heterogeneity may influence nitrogen solubility and the stability of the γN phase [12,13]. Severe plastic deformation (SPD) is widely considered an effective approach for modifying the defect structure of metallic materials [14]. Such treatment leads to grain refinement to an ultrafine-grained or nanocrystalline state, accompanied by an increase in dislocation density and the accumulation of stored deformation energy [13]. In metastable austenitic steels, partial deformation-induced martensitic transformation (γ → α′) may also occur, further increasing the density of structural defects [15]. Among SPD methods, ultrasonic nanocrystalline surface modification UNSM and vibro-impact mechanical treatment (VIMT) represent two fundamentally different approaches [16,17]. UNSM produces a gradient nanostructured surface layer (10–50 μm) with a high density of defects and compressive residual stresses [17], whereas vibro-impact mechanical treatment leads to the formation of a nonequilibrium structure with a high density of defects and stored deformation energy but with a less controlled deformation character [18]. In recent years, increasing attention has been paid to hybrid surface treatment approaches that combine severe plastic deformation with thermochemical processes, such as nitriding, to enhance the surface properties of metallic materials [19,20,21]. These combined treatments promote the formation of a highly defected near-surface layer, which significantly accelerates diffusion processes and improves the efficiency of subsequent modification steps. Despite these advances, it remains unclear how different defect structures formed by UNSM and vibro-impact mechanical treatment (VIMT) influence the kinetics of nitrogen diffusion, the thickness of the nitrided layer, and the resulting mechanical properties of the material. AISI 321 stainless steel is widely used in power engineering, chemical processing, and high-temperature applications, where components are subjected to friction, wear, and cyclic loading. In such conditions, improved surface hardness and structural stability are critical for ensuring long-term performance. Thus, the present study investigates the effect of different methods of preliminary plastic deformation on the formation of the nitrided layer during ion-plasma nitriding of 12Kh18N10T stainless steel. It is assumed that different types of preliminary treatment (UNSM and vibro-impact ball mechanical treatment) may distinctly influence nitrogen diffusion behavior and phase formation. The aim of this work is a comparative investigation of the effect of these methods on the mechanical properties of 12Kh18N10T stainless steel after ion-plasma nitriding.

## 2. Materials and Methods

In this study, samples made of austenitic stainless steel 12Kh18N10T (foreign analogue—AISI 321) were used. The specimens were prepared from a rod with a diameter of 50 mm and a thickness of 5 mm. The chemical composition of the steel (wt.%) was as follows: Fe—70.67; Cr—18.57; Ni—9.70; Mn—0.19; Ti—0.21; Si—0.23; Cu—0.29; C—0.12; P—0.01; S—0.01. For each treatment condition (VIMT + IPN and UNSM + IPN), two specimens (*n* = 2) were prepared and analyzed. The results are presented as average values obtained from repeated measurements.

The selection of AISI 321 (12Kh18N10T), instead of the more widely used AISI 304, is justified by its superior stability at elevated temperatures and enhanced resistance to intergranular corrosion due to titanium stabilization. These characteristics make AISI 321 particularly suitable for applications involving thermal exposure and aggressive environments, where improved surface performance is required.

Prior to mechanical treatment, the substrates were sequentially ground using SiC papers with grit sizes ranging from 200 to 2500 and subsequently mechanically polished to obtain a smooth and uniform surface. After grinding, the samples were rinsed with distilled water to remove abrasive particles and surface contaminants.

To investigate the influence of different preliminary treatment methods, the specimens were divided into two groups. The designations and processing conditions of the samples are summarized in Table 1. The first group of specimens was subjected to vibro-impact mechanical treatment using a universal vibration stand SVU-2 (Vibromash, Ust-Kamenogorsk, Kazakhstan) [22], which represents a single-mass over-resonance type installation. A detailed description of the design and operating principle of this system is provided in the work of Rakhadilov B. et al. [22]. The treatment was carried out using steel balls with a diameter of 6 mm at a vibration frequency of 30 Hz for 15 min. The working chamber of the installation was filled with balls to approximately 80% of its volume, ensuring intensive impact of the balls on the surface of the specimens.

**Figure 1:**
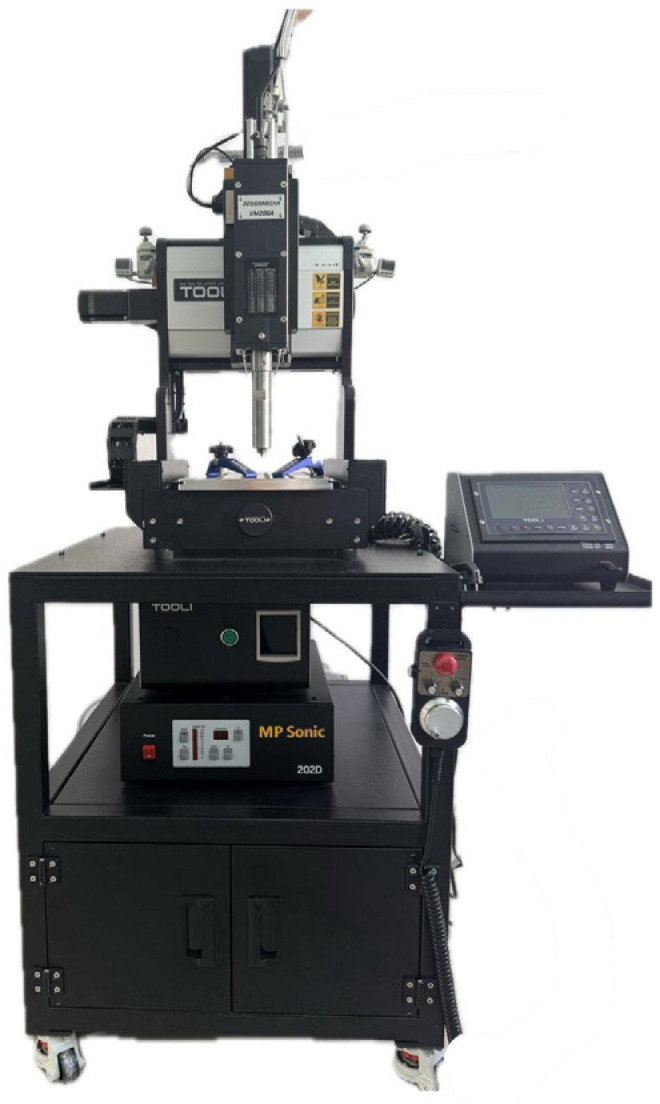
Setup for ultrasonic nanocrystalline surface modification (UNSM).

The second group of specimens was subjected to ultrasonic nanocrystalline surface modification (UNSM), a surface severe plastic deformation technique based on the action of high-frequency impacts of a spherical indenter on the treated surface (Figure 1). As a result of the treatment, a nanocrystalline surface layer is formed, the density of crystal lattice defects increases, and the stored energy in the surface layers of the material rises, which contributes to the intensification of subsequent diffusion processes [15,17].

After preliminary mechanical treatment, all specimens were subjected to ion-plasma nitriding (IPN). The process was carried out using a specialized laboratory installation LDMC-20 (Model LDMC-20, Tianman Industrial Furnace, Tianjin, China). A detailed description of the installation and its operating principle is provided in the work of Rakhadilov et al. [23]. The nitriding process was carried out using ammonia (NH_3_) as the working gas, which serves as a source of active nitrogen species during plasma-assisted dissociation. Nitriding was performed at a temperature of 500 °C for 10 h at a working pressure of 250 Pa. The selected processing parameters ensured the formation of a diffusion nitrided layer on the steel surface. To ensure accurate temperature control during nitriding, a calibration procedure was performed prior to the experiments. Although the temperature sensor was located inside the vacuum chamber and was not directly attached to the sample, its readings were verified using an external infrared pyrometer. During calibration, the cathode was heated to a predetermined temperature, and the thermocouple readings were compared with the pyrometer measurements. The deviation between the thermocouple readings and the actual surface temperature did not exceed ±5 °C. This confirms that the temperature recorded by the chamber sensor reliably reflects the actual temperature of the samples during the ion-plasma nitriding process [24].

The selection of VIMT and UNSM is based on the need to compare two fundamentally different mechanisms of severe plastic deformation. VIMT provides stochastic impact deformation induced by multiple ball collisions, while UNSM produces controlled high-frequency surface impacts resulting in a gradient nanostructured layer. This approach allows a meaningful comparison of how different defect structures influence nitrogen diffusion kinetics and nitrided layer formation.

Hardness measurements were carried out using a FISCHERSCOPE HM2000 measurement (Helmut Fischer GmbH, Sindelfingen, Germany) system in accordance with the DIN EN ISO 14577-1 standard [25]. A diamond Vickers indenter with a square pyramidal geometry and an angle of 136° between opposite faces was used. The tests were performed at a maximum load of 100 mN with a linear loading time of 15 s and a dwell time of 5 s at the maximum load. The indentation depth did not exceed 10% of the thickness of the hardened layer, which corresponds to the generally accepted criterion (“10% rule”) for instrumented indentation and ensures the reliability of the obtained hardness values.

The surface roughness (Ra) of the treated samples was measured using a profilometer (SSR300+, Shenzhen, China). The measurements were performed using a cut-off length of 0.8 mm and an evaluation length of 2.4 mm. For each sample, ten measurements were performed, and the average roughness value was calculated.

The phase composition was determined using an X-ray diffractometer X’Pert Pro (PANalytical, Amsterdam, The Netherlands) with Cu-Kα radiation operating at a voltage of 40 kV and a current of 30 mA. The scanning parameters were set in the range of 35° < 2θ < 85° with a step size of 0.02° and a counting time of 5 s per step.

For microstructural analysis, cross-sections of the samples were prepared. Initially, mechanical cutting was performed using silicon carbide cutting wheels. The samples were then ground and polished using diamond paste to obtain a smooth surface free of mechanical deformation. Chemical etching was carried out in a 10% oxalic acid solution. The microstructure was characterized using a scanning electron microscope (SEM) TESCAN VEGA (Tescan, Brno, Czech Republic).

Scratch testing was performed using a Revetest scratch tester (RST 300, Anton Paar, Graz, Austria) under progressive loading conditions. A Rockwell-type diamond indenter with a tip radius of 200 μm was used. The normal load was linearly increased from 0.5 N to 30 N at a loading rate of 44.2 N/min. The scratch length was 4 mm, and the sliding speed was 6 mm/min. During the test, the normal load (Fn), tangential force (Ft), and acoustic emission (AE) signals were continuously recorded. Friction tests were carried out on a universal tribological tester TRB3 (Anton Paar GmbH, Graz, Austria) using the “ball-on-disk” method in accordance with ASTM G99 standard [26].

## 3. Results

### 3.1. Phase Composition and X-Ray Diffraction Analysis

**Figure 2:**
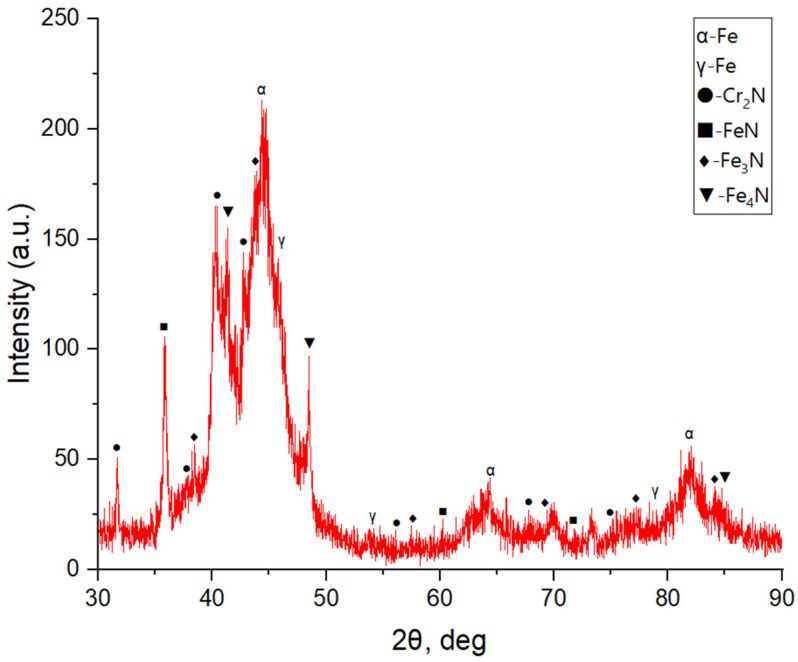
X-ray diffraction (XRD) patterns of 12Kh18N10T austenitic stainless steel after vibro-impact mechanical treatment (VIMT) followed by ion-plasma nitriding (IPN).

Figure 2 shows the X-ray diffraction (XRD) patterns of 12Kh18N10T austenitic stainless steel after vibro-impact mechanical treatment (VIMT) followed by ion-plasma nitriding (IPN). The diffraction patterns indicate that the austenitic γ-phase with a face-centered cubic (FCC) crystal lattice is retained in the nitrided surface layer. The main diffraction peaks correspond to the γ-Fe reflections ((111), (200), and (220)). In addition, peaks corresponding to α-Fe are observed, which may be associated with partial deformation-induced transformation of austenite into martensite during mechanical treatment [15]. Reflections corresponding to nitride phases, such as Cr_2_N and Fe_3_N, are also detected, indicating the formation of a nitrogen-enriched surface layer during ion-plasma nitriding. The presence of these phases is attributed to nitrogen diffusion into the near-surface region and its interaction with alloying elements [4,7].

A shift in the γ-phase diffraction peaks toward lower 2θ angles is observed compared with the initial state. This shift may indicate an increase in the lattice parameter due to the incorporation of nitrogen atoms into the interstitial sites of austenite. Such behavior is commonly associated with nitrogen supersaturation and may suggest the formation of expanded austenite (γN or S-phase) [4,5,6]. However, it should be noted that the presence of expanded austenite cannot be conclusively confirmed based solely on XRD data due to peak overlap and the simultaneous formation of nitride phases [2,4].

**Figure 3:**
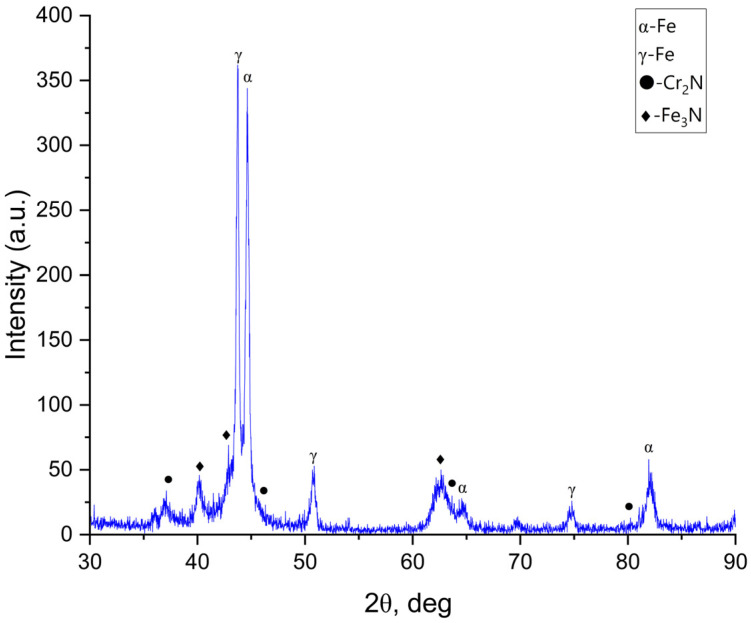
X-ray diffraction (XRD) patterns of 12Kh18N10T stainless steel after ultrasonic nanocrystalline surface modification (UNSM) followed by ion-plasma nitriding (IPN).

Figure 3 presents the XRD patterns of 12Kh18N10T stainless steel after ultrasonic nanocrystalline surface modification (UNSM) followed by ion-plasma nitriding. Similar to the VIMT-treated sample, the diffraction patterns are dominated by γ-Fe reflections, indicating that the austenitic structure is preserved after treatment. Peaks corresponding to α-Fe are also detected, which may result from deformation-induced martensitic transformation under severe plastic deformation conditions [13,15]. Reflections of nitride phases, including Cr_2_N and Fe_3_N, are observed in the diffraction pattern. This confirms the formation of a nitrogen-enriched diffusion layer [4,7]. Compared with the VIMT + IPN sample, the UNSM + IPN sample exhibits more pronounced peak broadening and slight peak shifts. The broadening of diffraction peaks is associated with a reduction in the size of coherent scattering regions and an increase in dislocation density induced by severe plastic deformation [13,14,24]. The observed shift in γ-phase peaks toward lower 2θ angles may indicate lattice expansion caused by nitrogen incorporation. This behavior may suggest a higher degree of nitrogen supersaturation in the UNSM-treated sample [4,11]. Thus, preliminary treatment by the UNSM method leads to the formation of a more defective and nanostructured near-surface layer, which enhances nitrogen diffusion during ion-plasma nitriding [9,11]. The XRD results, in combination with SEM and EDS analysis, indicate the formation of a nitrogen-enriched diffusion layer, while the presence of expanded austenite is suggested but cannot be conclusively confirmed.

### 3.2. Microstructure and EDS Analysis

**Figure 4:**
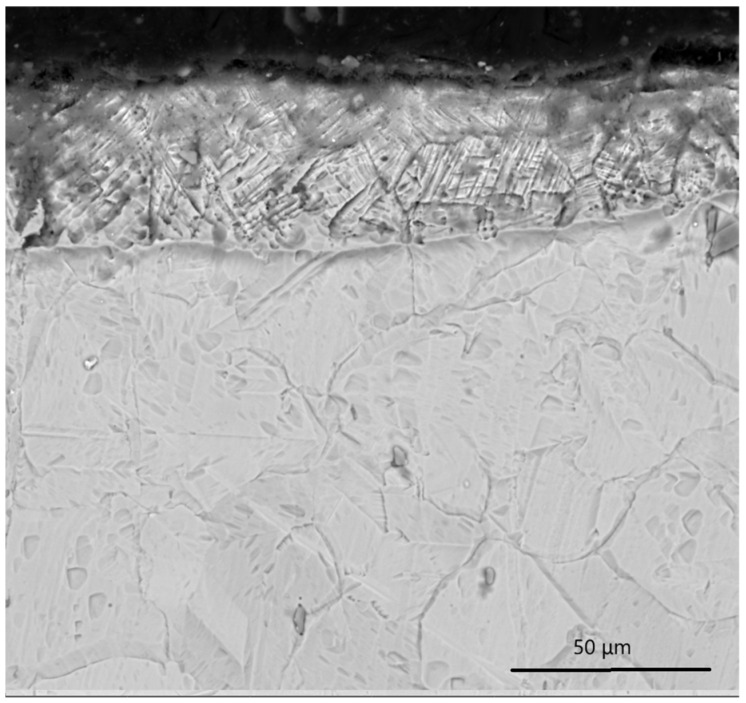
SEM cross-sectional image of the nitrided layer formed on 12Kh18N10T stainless steel after vibro-impact ball mechanical treatment (VIMT) followed by ion-plasma nitriding (IPN).

**Figure 5:**
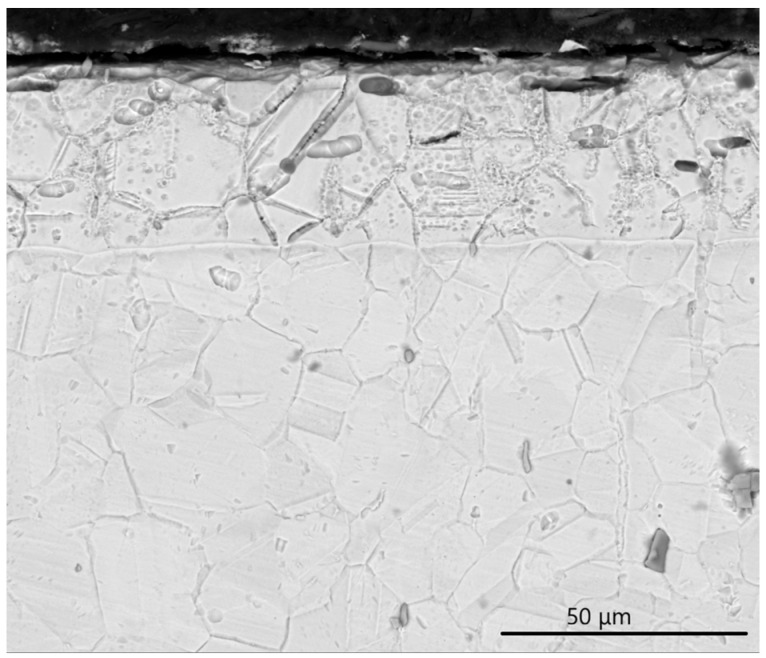
SEM cross-sectional image of the nitrided layer formed on 12Kh18N10T stainless steel after ultrasonic nanocrystalline surface modification (UNSM) followed by ion-plasma nitriding (IPN).

Figure 4 and Figure 5 present SEM micrographs of cross-sections of 12Kh18N10T stainless steel after ion-plasma nitriding performed at 500 °C for 10 h following different types of preliminary mechanical treatment. Figure 4 shows the microstructure of the sample after vibro-impact mechanical surface treatment (VIMT) followed by ion-plasma nitriding.

In the near-surface region, a diffusion nitrided layer is formed with a relatively well-defined interface between the modified layer and the substrate. The nitrided layer is characterized by a dense structure without signs of delamination, pore formation, or macroscopic defects. The transition zone is rather sharp, indicating a diffusion-controlled mechanism of layer formation under a moderate density of defects generated by the preliminary vibro-impact treatment [4,10]. The microstructure of the sample after ultrasonic nanocrystalline surface modification (UNSM) followed by ion-plasma nitriding is shown in Figure 5. Compared with the VIMT-treated sample, a more developed diffusion zone is observed, characterized by a smoother transition between the modified layer and the substrate. This indicates a higher diffusion activity and a more pronounced gradient structure of the nitrided layer. The elemental composition of the near-surface region is presented in Table 2. A comparison between the VIMT + IPN and UNSM + IPN samples shows that the UNSM-treated sample exhibits a significantly higher nitrogen concentration (15.56 wt.% compared to 4.35 wt.%). This clearly indicates enhanced nitrogen diffusion and the formation of a more developed nitrided layer after UNSM treatment.

The results of energy-dispersive spectroscopy (EDS) confirm the formation of a nitrogen-enriched surface layer. The nitrogen concentration in the UNSM + IPN sample reaches its maximum in the near-surface region and gradually decreases with increasing distance from the surface. This elemental distribution indicates the formation of a gradient diffusion layer, which is typical for nitrogen diffusion processes in austenitic stainless steels [4,11].

It is well known that severe plastic deformation leads to grain refinement to an ultrafine-grained or nanocrystalline state, accompanied by an increase in dislocation density and the accumulation of stored deformation energy [13]. Crystal lattice defects, including grain boundaries, sub-boundaries, and dislocations, act as accelerated diffusion paths, thereby increasing the nitrogen diffusion coefficient and promoting the rapid formation of expanded austenite (γN phase) [4,11,13,27].

Thus, the more pronounced diffusion layer observed in the UNSM-treated sample can be attributed to the higher density of crystal lattice defects, which enhance nitrogen diffusion during ion-plasma nitriding.

The thickness of the nitrided layer was estimated from SEM cross-sectional images. The VIMT + IPN sample exhibits a layer thickness of approximately 15–20 μm, while the UNSM + IPN sample shows a thicker modified layer of approximately 30–40 μm. It should be noted that these values are approximate due to the limitations of SEM-based measurements and the gradual nature of the diffusion layer. The obtained results are in good agreement with the XRD analysis and microhardness measurements, suggesting a synergistic effect of preliminary plastic deformation and ion-plasma nitriding.

### 3.3. Microhardness

**Figure 6:**
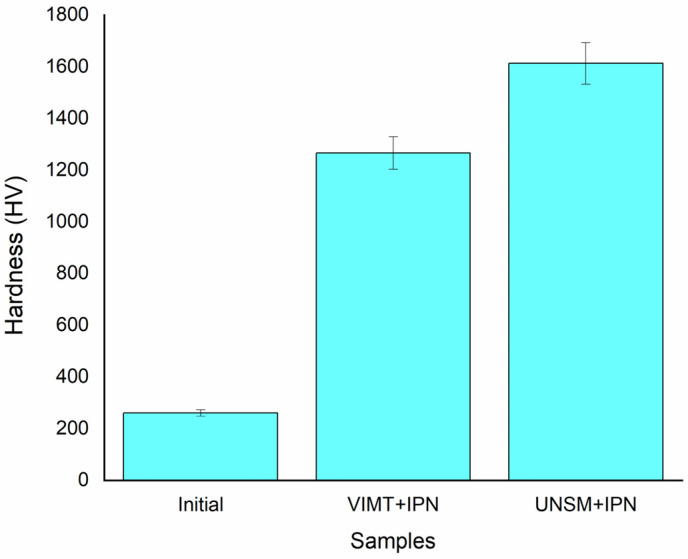
Microhardness of 12Kh18N10T stainless steel in the initial state and after different types of preliminary treatment followed by ion-plasma nitriding.

The microhardness results of 12Kh18N10T stainless steel in the initial state and after different types of preliminary treatment followed by ion-plasma nitriding are presented in Figure 6. The microhardness of the initial sample was 260 HV, which corresponds to typical values for austenitic stainless steels with a face-centered cubic (FCC) structure [28].

After vibro-impact mechanical treatment followed by ion-plasma nitriding, the hardness increased to 1266 HV, which is more than 4.8 times higher than the initial value. The maximum microhardness was obtained in the samples subjected to ultrasonic nanocrystalline surface modification (UNSM) followed by ion-plasma nitriding, reaching 1613 HV, which exceeds the initial hardness by more than six times. The increase in hardness after nitriding is attributed to the formation of a nitrogen-enriched diffusion layer consisting predominantly of expanded austenite (γN or S-phase), which is characterized by a supersaturated solid solution of nitrogen in the FCC lattice [2,6,7,11]. The incorporation of nitrogen atoms into the interstitial sites of the FCC lattice causes elastic lattice expansion and the development of high internal stresses, which increases the resistance to plastic deformation [13]. An additional contribution to strengthening is provided by the solid-solution strengthening mechanism due to nitrogen supersaturation in austenite. The differences in hardness between the samples after vibro-impact mechanical treatment (VIMT) and UNSM are related to the nature of the formed defect structure. Severe plastic deformation induced by UNSM leads to grain refinement to an ultrafine-grained and nanocrystalline state, accompanied by a significant increase in dislocation density [14,15,16].

The increased density of crystal lattice defects creates accelerated diffusion pathways, which increases the effective nitrogen diffusion coefficient and promotes the formation of a thicker and more nitrogen-enriched γN layer [11,13,26]. In the case of vibro-impact mechanical treatment, a nonequilibrium structure with a high density of defects is also formed [19,20]; however, the deformation is predominantly volumetric rather than surface-localized, resulting in a less pronounced gradient structure and a lower concentration of defects in the near-surface region directly involved in the nitriding process [15]. In addition, compressive residual stresses generated during UNSM may contribute to the stabilization of expanded austenite and increase its resistance to decomposition [2,6,15]. As a result, a more pronounced hardened layer is formed, which is reflected in the higher microhardness values.

Thus, the increase in hardness is determined by the combined effect of solid-solution strengthening, residual stresses, and the specific defect architecture formed by different methods of preliminary plastic deformation [11,13,26].

### 3.4. Scratch Resistance and Adhesion Behavior

**Figure 7:**
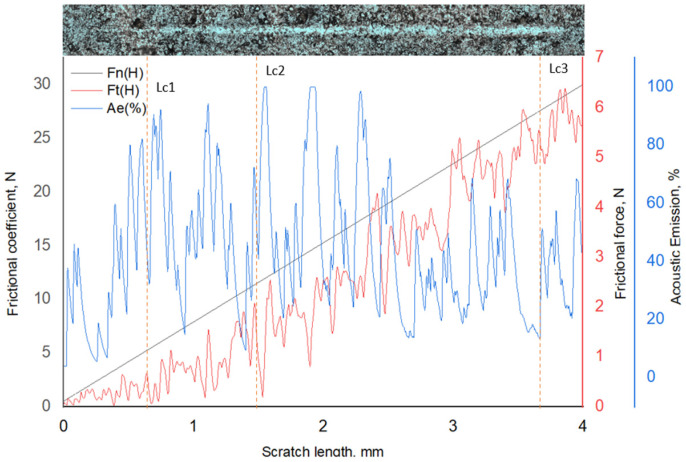
Scratch test results of 12Kh18N10T stainless steel after vibro-impact ball mechanical treatment (VIMT) followed by ion-plasma nitriding (IPN).

Figure 7 presents the results of progressive scratch testing of 12Kh18N10T stainless steel after vibro-impact mechanical treatment (VIMT) followed by ion-plasma nitriding (IPN). An optical image of the scratch track is presented above the graph, indicating the locations where the critical loads (Lc1–Lc5) were determined. During the test, the normal load (Fn), tangential friction force (Ft), and acoustic emission (AE) signal were continuously recorded. The critical loads were primarily determined based on characteristic changes in the slope and sharp fluctuations of the Ft curve, while the AE signal was used as an additional indicator of damage events.

The first critical load, Lc1 (Ft ≈ 0.7 N), corresponds to the initiation of the first local damage within the nitrided surface layer. This is confirmed by a change in the character of the Ft dependence and the appearance of instability in the friction signal. The second critical load, Lc2 (Ft ≈ 1.45 N), is associated with the development of damage and the transition to a more unstable sliding regime, which is manifested by an increase in the amplitude of Ft fluctuations and enhanced acoustic emission.

The third critical load, Lc3 (Ft ≈ 3.82 N), corresponds to the stage of pronounced failure of the hardened layer. In this region, a significant increase and instability of Ft are observed, accompanied by intense AE bursts, which are typical for progressive cracking and failure of the layer under increasing contact load.

With further increase in load (Lc4–Lc5), the continued rise in Ft reflects further degradation of the near-surface zone and the increasing contribution of plastic deformation of the substrate after substantial destruction of the nitrided layer.

In general, the relatively low values of the critical loads determined from the Ft curve (Lc1–Lc3) for the VIMT + IPN sample indicate an earlier transition to the failure regime compared with the samples after UNSM treatment, which may be associated with the structural state of the surface and the characteristics of the defect structure formed during preliminary treatment [13,15].

**Figure 8:**
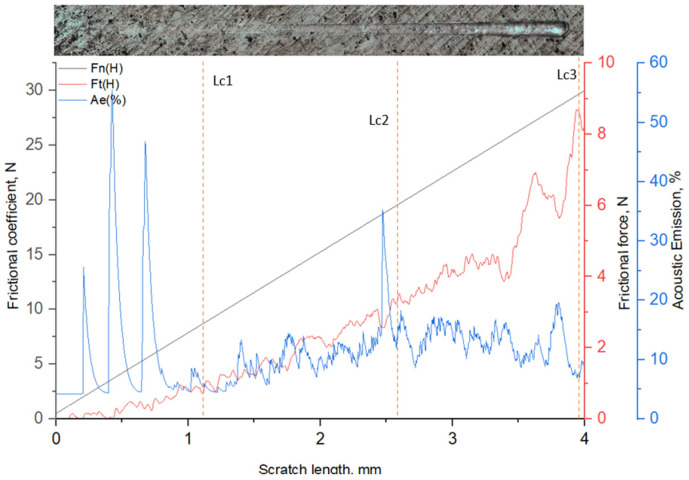
Scratch test results of 12Kh18N10T stainless steel after ultrasonic nanocrystalline surface modification (UNSM) followed by ion-plasma nitriding (IPN).

Figure 8 presents the results of progressive scratch testing of 12Kh18N10T stainless steel after UNSM followed by ion-plasma nitriding. During the test, the normal load (Fn), tangential friction force (Ft), and acoustic emission (AE) signal were recorded. The critical loads were mainly determined based on characteristic changes and jumps in the Ft curve, supported by analysis of the AE signal.

The first critical load, Lc1 (Ft ≈ 1.29 N), corresponds to the initiation of the first local damage in the nitrided layer. Compared with the VIMT + IPN sample, the Lc1 value is higher, indicating greater resistance of the surface to initial shear damage. The second critical load, Lc2 (Ft ≈ 2.89 N), is associated with the development of damage and the transition to a more pronounced unstable friction regime. An increase in the amplitude of Ft fluctuations and enhanced acoustic emission is observed, indicating progressive cracking of the hardened layer.

The third critical load, Lc3 (Ft ≈ 5.95 N), corresponds to the stage of pronounced failure of the diffusion layer. In this region, a significant increase in Ft and strong signal fluctuations are recorded, which are characteristic of intensive damage development and partial destruction of the near-surface zone.

With further load increase (Lc4 ≈ 8.82 N; Lc5 ≈ 12.17 N), the continued growth of Ft reflects the development of plastic deformation involving the substrate after degradation of the nitrided layer.

In general, the higher values of the critical loads determined from the Ft curve (Lc1–Lc3), compared with the VIMT + IPN sample, indicate increased resistance of the hardened layer to progressive shear failure. Such behavior is attributed to a more pronounced structural gradient, higher microhardness, and the formation of a more stable γN layer [4,6,15]. Thus, the scratch test confirms that preliminary ultrasonic nanocrystalline surface modification not only increases microhardness but also significantly enhances the resistance to local damage and delamination of the hardened layer [15,17].

### 3.5. Surface Roughness

The results of surface roughness measurements of the samples after different types of preliminary treatment followed by ion-plasma nitriding are presented in Figure 8. For the quantitative evaluation of the surface condition, the average arithmetic roughness (Ra) and the maximum height of the roughness profile (Rz) parameters were used.

**Figure 9:**
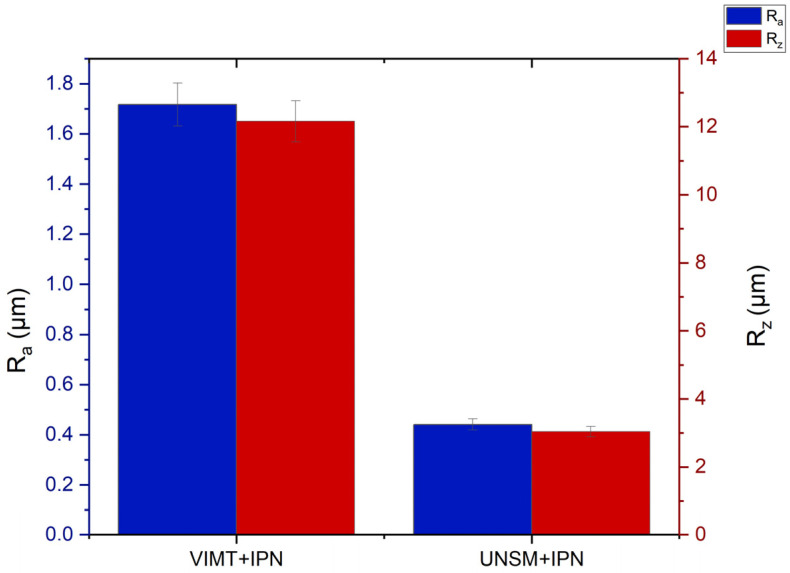
Surface roughness parameters (Ra and Rz) of 12Kh18N10T stainless steel after different types of preliminary surface treatment followed by ion-plasma nitriding.

As shown in Figure 9, the sample subjected to vibro-impact mechanical treatment followed by ion-plasma nitriding (VIMT + IPN) is characterized by relatively high roughness values. The Ra parameter reaches approximately 1.7 μm, while the Rz parameter is about 12 μm, indicating the formation of a pronounced and non-uniform surface relief. Such surface morphology can be explained by the stochastic nature of repeated impacts of steel balls during vibro-impact treatment, leading to the formation of localized plastic deformation zones and an uneven surface topography [15,19]. In contrast, the sample subjected to ultrasonic nanocrystalline surface modification followed by ion-plasma nitriding (UNSM + IPN) demonstrates significantly lower roughness values. The Ra parameter decreases to approximately 0.4 μm, while the Rz value is reduced to about 3 μm.

The reduction in surface roughness indicates that the UNSM process promotes the formation of a smoother and more uniform surface compared with vibro-impact treatment. The improvement in surface quality after UNSM can be explained by the high-frequency controlled impacts of a spherical indenter, which induce intensive plastic deformation of the surface in a localized and controlled manner. This process leads to surface smoothing, grain refinement, and the formation of a more homogeneous nanostructured surface layer.

Similar effects of surface smoothing and microstructure refinement after UNSM treatment have been reported in several previous studies [15,17]. Thus, the obtained results indicate that preliminary treatment using the UNSM method provides not only grain refinement and an increase in hardness, but also a significant reduction in surface roughness and improved surface uniformity. The surface roughness values correspond to samples after combined treatment. Vibro-impact ball mechanical treatment generally increases surface roughness, whereas ultrasonic nanocrystalline surface modification leads to a smoother and more uniform surface [17].

Such surface characteristics are favorable for subsequent tribological performance and may contribute to improved wear resistance, as reported in recent studies on surface-modified stainless steels [21,26,29]. It should be noted that the surface roughness values presented in this study correspond to samples after combined treatment (pretreatment followed by ion-plasma nitriding). The roughness after VIMT and UNSM treatments alone was not evaluated in the present work. However, according to the known mechanisms of these methods, vibro-impact mechanical treatment (VIMT) typically increases surface roughness due to random impact deformation, whereas ultrasonic nanocrystalline surface modification (UNSM) tends to produce a smoother and more uniform surface as a result of controlled plastic deformation.

### 3.6. Tribological Behavior

The tribological behavior of 12Kh18N10T stainless steel after different types of preliminary treatment followed by ion-plasma nitriding was evaluated under dry sliding conditions.

**Figure 10:**
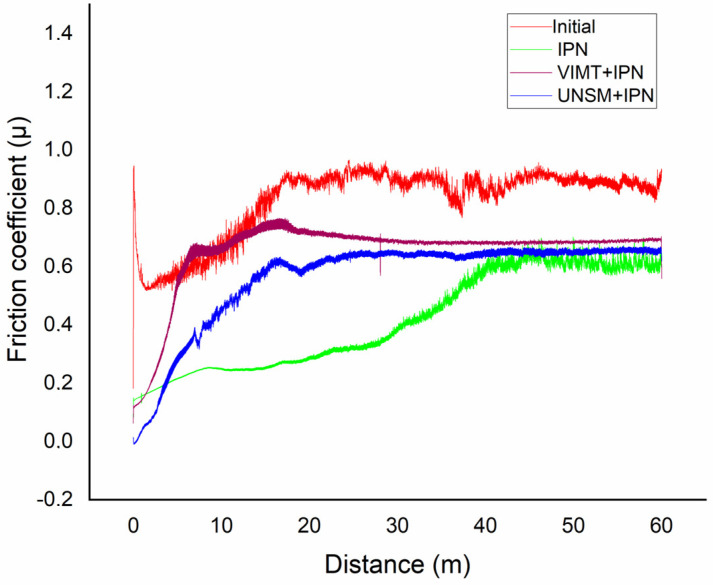
Variation in the friction coefficient of 12Kh18N10T stainless steel after different types of preliminary treatment followed by ion-plasma nitriding.

The variation in the friction coefficient as a function of sliding time is presented in Figure 10. For all samples, the friction coefficient initially increases during the running-in stage, followed by a transition to a steady-state regime. This behavior is typical for sliding contacts and is associated with the gradual adaptation of the contact surfaces. The VIMT + IPN sample exhibits a higher and less stable friction coefficient compared with the UNSM + IPN sample, indicating a more pronounced fluctuation in the contact conditions and less stable surface behavior. In contrast, the UNSM + IPN sample demonstrates a lower and more stable friction coefficient, which can be attributed to the formation of a smoother and more homogeneous surface layer, as well as higher microhardness and a more uniform defect structure.

The improved tribological performance of the UNSM-treated sample is associated with its refined microstructure, increased hardness, and reduced surface roughness, which together contribute to enhanced wear resistance and stability under sliding conditions [13,15,17,29]. Thus, the results confirm that preliminary ultrasonic nanocrystalline surface modification significantly improves the tribological characteristics of ion-plasma nitrided 12Kh18N10T stainless steel under the selected testing conditions.

## 4. Conclusions

The present study demonstrates that preliminary severe plastic deformation significantly affects the efficiency of ion-plasma nitriding and the formation of a nitrided layer in 12Kh18N10T stainless steel. It was shown that different deformation mechanisms lead to distinct defect structures, which directly influence nitrogen diffusion behavior and the resulting surface properties.

The main findings of the present study can be summarized as follows:

- Under the selected processing conditions, ultrasonic nanocrystalline surface modification (UNSM) promotes the formation of a thicker and more uniform diffusion layer compared with vibro-impact ball mechanical treatment (VIMT).
- The UNSM-treated samples exhibit higher microhardness and improved resistance to surface damage.
- The results highlight the importance of controlling the defect structure prior to nitriding as a key factor in enhancing the performance of austenitic stainless steels.
- Tribological tests showed that the UNSM + IPN samples exhibit lower and more stable friction behavior, indicating improved wear resistance under the selected testing conditions.

These findings contribute to the understanding of hybrid surface treatment approaches and may be useful for optimizing surface strengthening technologies for engineering applications.

## Figures and Tables

**Table 1:** Sample designations used in the study.

No of Sample	Treatment	Processing
1 (VIMT + IPN)	Vibro-impact ball mechanical treatment + ion-plasma nitriding	Vibration frequency 30 Hz; steel ball diameter 6 mm; treatment time 15 min; chamber filling ≈ 80%
2 (UNSM + IPN)	Ultrasonic nanocrystalline surface modification + ion-plasma nitriding	UNSM parameters: amplitude 30 μm, static load 30 N, treatment at room temperature (RT)

**Table 2:** Comparative elemental composition (wt.%) of the near-surface region of 12Kh18N10T stainless steel after VIMT + IPN and UNSM + IPN treatments.

Sample	N Weight,%	O Weight,%	Si Weight,%	Ti Weight,%	Cr Weight,%	Mn Weight,%	Fe Weight,%	Ni Weight,%
(VIMT + IPN)	4.35	6.21	0.48	0.54	14.69	0.64	54.79	7.08
(UNSM + IPN)	15.56	4.76	0.35	0.44	13.61	0.65	58.37	6.26

## Data Availability

The original contributions presented in the study are included in this article; further inquiries can be directed to the corresponding author.
